# Vocal Age Disguise: The Role of Fundamental Frequency and Speech Rate and Its Perceived Effects

**DOI:** 10.3389/fpsyg.2016.01814

**Published:** 2016-11-21

**Authors:** Sara Skoog Waller, Mårten Eriksson

**Affiliations:** Department of Social Work and Psychology, Faculty of Health and Occupational Studies, University of GävleGävle, Sweden

**Keywords:** age disguise, voice disguise, age estimation, fundamental frequency, speech rate, voice manipulation, deception, age perception

## Abstract

The relationship between vocal characteristics and perceived age is of interest in various contexts, as is the possibility to affect age perception through vocal manipulation. A few examples of such situations are when age is staged by actors, when ear witnesses make age assessments based on vocal cues only or when offenders (e.g., online groomers) disguise their voice to appear younger or older. This paper investigates how speakers spontaneously manipulate two age related vocal characteristics (*f*_0_ and speech rate) in attempt to sound younger versus older than their true age, and if the manipulations correspond to actual age related changes in *f*_0_ and speech rate (Study 1). Further aims of the paper is to determine how successful vocal age disguise is by asking listeners to estimate the age of generated speech samples (Study 2) and to examine whether or not listeners use *f*_0_ and speech rate as cues to perceived age. In Study 1, participants from three age groups (20–25, 40–45, and 60–65 years) agreed to read a short text under three voice conditions. There were 12 speakers in each age group (six women and six men). They used their natural voice in one condition, attempted to sound 20 years younger in another and 20 years older in a third condition. In Study 2, 60 participants (listeners) listened to speech samples from the three voice conditions in Study 1 and estimated the speakers’ age. Each listener was exposed to all three voice conditions. The results from Study 1 indicated that the speakers increased fundamental frequency (*f*_0_) and speech rate when attempting to sound younger and decreased *f*_0_ and speech rate when attempting to sound older. Study 2 showed that the voice manipulations had an effect in the sought-after direction, although the achieved mean effect was only 3 years, which is far less than the intended effect of 20 years. Moreover, listeners used speech rate, but not *f*_0_, as a cue to speaker age. It was concluded that age disguise by voice can be achieved by naïve speakers even though the perceived effect was smaller than intended.

## Introduction

The human voice changes from childhood and throughout an individual’s lifespan because of biochemical and physiological changes affecting the speech mechanism, as well as the result of sociolinguistic influence. Regularities in this variation allow listeners to make fairly accurate assessments of the speaker’s age from his or her voice and may also be used by speakers to give the impression of being younger or older than s/he actually is. Listeners generally rely on several voice parameters in their age estimates. For example, jitter, shimmer, noise and tremor, have been found to influence estimation of speaker age (Brückl and Sendlmeier, 2003; Schötz, 2006; Harnsberger et al., 2008), yet fundamental frequency (*f*_0_) and speech rate are widely accepted as being particularly important (e.g., Linville, 1996; Harnsberger et al., 2008; Skoog Waller et al., 2015). However, it is unknown if *f*_0_ and speech rate are actually modulated when speakers try to sound either younger or older, and if so, whether manipulations in *f*_0_ and speech rate correspond to actual age related changes in the same voice parameters.

Speech rate decreases with age for both female and male speakers (e.g., Harnsberger et al., 2008; Skoog Waller et al., 2015) while changes in *f_0_* look different in male speakers compared to female speakers. For female speakers, *f_0_* does not change much until the menopause after which a drop occurs. In contrast, *f_0_* in aging male speakers follows a U-function being lowest between 40 and 50 years, reaching the level of 20–30 years at age 60–70 years, and then continues to rise (see review by Linville, 1996).

Listeners are relatively accurate in estimating speaker age. Several studies (Shipp and Hollien, 1969; Huntley et al., 1987; Neiman and Applegate, 1990; Braun, 1996; Brückl and Sendlmeier, 2003) have reported robust correlations (0.70–0.90) between estimated speaker age and the chronological age of the speakers. One factor leading to unprecise estimations is a bias toward the mean population age. Older speakers are regularly estimated as younger than they actually are while younger speakers are estimated as older than they are (see Shipp and Hollien, 1969; Hollien and Tolhurst, 1978; Huntley et al., 1987; Braun, 1996; Braun and Cerrato, 1999; Brückl and Sendlmeier, 2003; Skoog Waller et al., 2015).

Individuals may want to sound younger or older for numerous reasons. Actors on stage, in film and other media incessantly make portrayals in relation to the spectrum of age that draw on beliefs about vocal aging (Marshall and Lipscomb, 2010). In this context it is of value to understand how certain voice characteristics are related to perceived age.

For young asylum seekers age estimation is often a more fateful matter because special laws regulate the rights for admittance of minors. However, the age estimations are based on uncertain methods (Sauer et al., 2016) and the final decision is an overall assessment from various sources.

In the daily life of most people, age assessments are made in judgements and descriptions of speakers based on their voices. Such descriptions are also frequently made by victims and witnesses of crime who have encountered perpetrators under poor visual conditions (Yarmey et al., 1996; Yarmey, 2001, 2004). Testimonies may be based on observations made in the dark or the perpetrator may have hindered the victim or witness from seeing him by using force or by wearing some kind of mask. Some descriptions are based solely on acoustic information, e.g., when a perpetrator have not been observed visually but heard over the phone. Witnesses often provide assessments about the age of unknown perpetrators and such information can indeed be valuable in crime investigations. It is therefore important for law enforcers to have knowledge about the grounds on which age estimations are made (such as the relation between specific voice parameters and age estimates) and how precise estimations can be expected to be.

In some forensic cases interception may be performed to provide voice recordings that can be used to identify criminals through forensic voice analysis. In other cases identification may be achieved by ear witnesses. In either case, voice identification is subject to error at a relatively high rate (Boë, 2000) and may often be further afflicted by the fact that criminals frequently disguise their voices in order to obstruct identification (Reich and Duke, 1979; Orchard and Yarmey, 1995; Boë, 2000; Neuhauser, 2008; Suneetha, 2013). Voice disguise can be performed in various ways, some of them with the help of electronic devices, others by using mechanical devices such as to put a handkerchief or the hand over the mouth or to pinch the nostrils (Perrot and Chollet, 2012). Künzel (2000) notes that 15–25% of the cases processed at the speaker identification section at BKA (the German Federal Police Office) contained common non-instrumental forms of vocal disguise including whisper, falsetto, quirky voice, imitation of dialect or foreign accent and age disguise with the intention to sound younger or older. Vocal age disguise is sometimes performed by online groomers when telephone contact is established between a groomer and a victim (e.g., Whittle et al., 2013).

In online grooming cases and similar crimes with the intention to abuse minors, the interest is primarily that of adults and older people to sound younger than their true age. However, there is reason to believe that older speakers are not as skilled as young speakers in modulating their voices due to physiological changes such as increased stiffness of vocal cord tissues. For example, older language learners’ usually have a more pronounced accent than younger ones (Stevens, 1999; Piske et al., 2001). Identification of voice parameters that are resistant to disguise would be of value for crime investigations.

Many recent studies on the effects of voice disguise concern the design of automatic speaker recognition systems to be used by the police (e.g., Perrot and Chollet, 2008; Zhang and Tan, 2008; Wu et al., 2014). However, such systems can never replace human perception in a witness situation because they require recording of the offenders’ voice, which is not always possible. Hence, effects of disguise on human perception will always be important. The effects of voice disguise on estimations of speaker age have previously been studied by Lass et al. (1982). Their study was based on young adults attempting to disguise their true age by sounding younger or older. Small differences in perceived age in the attempted directions were described although no inferential statistics were reported and no description of how (in terms of speech parameters) the voices were changed was given. No more recent study has investigated age disguise by vocal manipulation although the application of such research is more current today than 30 years ago due to recent phenomena such as online grooming.

The purpose of the present research was to extend the study of Lass et al. (1982) in several ways. In a first study (Study 1), we analyzed how women and men from various age groups spontaneously manipulate two of the most important age related voice parameters (*f*_0_ and speech rate) when instructed to disguise their voice to sound younger versus older and if the manipulations corresponded to actual age related changes in *f*_0_ and speech rate. The purpose of Study 2 was to examine the effects of vocal age disguise on perceived age. The study of Lass et al. (1982) was extended by including speakers from three age groups. Finally, the direct effects of *f*_0_ and speech rate on estimated age were examined in a cross-study analysis which also allowed us to investigate the relative contribution of each parameter.

## Study 1

The purpose of the first study was to investigate how female and male speakers from various age groups spontaneously manipulate *f*_0_ and speech rate when instructed to sound younger or older, and if the direction of the manipulations would correspond to the direction of actual age related changes in *f*_0_ and speech rate in female and male speakers. Speech rate decreases rather continuously with age in both female and male speakers (Harnsberger et al., 2008; Skoog Waller et al., 2015) while *f_0_* decreases notably after menopause in female speakers and follows a U-function in male speakers, being lowest during middle age (Linville, 1996). Thus, if vocal age disguise imitates actual vocal aging young men could be expected to speak with decreased *f_0_* to sound older, while middle aged and older men could be expected to increase their *f_0_* to sound older. To sound younger, on the other hand, middle aged men could be expected to increase *f_0_* while older men would be expected to decrease *f_0_.*

### Method

#### Participants

Voices from 36 speakers recruited from students and staff at the University of Gävle were used. The speakers were from three age groups: 20–25 years (*M* = 23.38 years, *SD* = 1.19), 40–45 years (*M* = 42.25 years, *SD* = 3.22) and 60–65 years (*M* = 62.67 years, *SD* = 1.87). There were 12 speakers in each age group (six women and six men). All speakers were non-smoking native speakers of Swedish. The studies reported in this paper were conducted in accordance with the declaration of Helsinki and the ethical guidelines given by the American Psychological Association. All participants (listeners and speakers) were adults and participated on informed consent. The listeners and the speakers signed an information agreement form. The experiment caused no harm to any part, the identity of the participants has been kept confidential, and no conflict of interest can be identified.

#### Material and Procedure

Speech samples of read speech with duration between 9 and 12 s were recorded in a quiet laboratory setting using a dynamic microphone placed 15 cm from the speaker’s mouth. Participation was rewarded with a movie ticket.

#### Voice Conditions

The speakers in the two older age groups were instructed to sound around 20 years younger in one condition, to use their natural voice in another condition and to sound around 20 years older in a third condition. We did not include speech samples from speakers in the youngest age group disguised to sound younger because the voice condition required the speakers to try to sound like children of 0–5 years of age which is quite another task than what was required in the other voice conditions. The youngest age group (20–25) was instructed to sound around 20 years older in one voice condition and to use their natural voice in another. Thus, in all 96 speech samples were obtained from the 36 speakers.

#### Analyses

The voices were edited in Audacity 1.2.6^1^. A standard feature was used to compress the dynamic range of the recordings, making the loudest parts softer while keeping the volume of the soft parts the same. The threshold value was set to -12 dB and the ratio was set to 2:1. The speech samples were then normalized for intensity by setting the maximum intensity of all the samples to the same value. The acoustic analyses on speech rate and fundamental frequency (*f*_0_) were made in Praat 5.4.06^2^, a software tool for analyzing, synthesizing and manipulating speech.

The data were computed and analyzed in SPSS 22.0 using mixed analysis of variance (ANOVA) models. *Post hoc* analyses were computed using the Bonferroni correction and the level of significance was set at 0.05. Because the study design did not include young speakers seeking to sound younger, two analyses were performed on fundamental frequency and speech rate respectively. The first included three voice conditions (young, natural, old) as a within-subject variable and two age groups (40–45, 60–65 years) as a between-subjects variable. The second analysis consisted of two voice conditions (natural, old) and three age groups (20–25, 40–45, 60–65 years). Sex of the speaker was included in both analyses because it is known that voices of women are higher than those of men (e.g., Titze, 1994). Mauchly’s test of sphericity indicated that the assumption of sphericity had not been violated (*W* > 0.90).

### Results and Discussion

#### Fundamental Frequency

Mean and standard deviation of *f_0_* for women and men over voice conditions and age groups are shown in **Table 1**. The mean *f_0_* was about the same for female voices between 20–25 and 40–45 years in the natural condition but lower for female voices 60–65 years. This change in female voices would be expected from the description of Linville (1996). However, *f_0_* for the male speakers in the natural condition followed an inverted U-function with the men 40–45 years at the peak which is contrary to the development described by Linville (1996). Yet, this comparison is between groups and might be due to individual variation. Importantly though, both female and male speakers raised *f_0_* when disguised as younger and lowered *f_0_* when disguised as older.

**Table 1:** Mean and standard deviation of voice parameters for 18 female voices and 18 male voices over conditions and age groups in Study 1.

	Disguise (younger)		Natural		Disguise (older)	
			
	*M*	*SD*	*M*	*SD*	*M*	*SD*
**Fundamental frequency (Hz)**						
**Women**						
20–25	–	–	199.67	19.99	189.72	26.55
40–45	210.45	38.17	198.97	21.60	183.19	20.22
60–65	200.17	21.31	190.76	8.36	178.97	21.25
**Men**						
20–25	–	–	112.82	13.99	109.98	18.58
40–45	138.12	25.68	122.24	15.99	109.36	19.04
60–65	116.04	15.44	113.61	10.63	109.28	15.21
**Speech rate (syllables/s)**						
**Women**						
20–25	–	–	4.21	0.41	3.66	0.24
40–45	4.71	0.45	4.08	0.53	3.29	0.89
60–65	4.13	0.50	3.54	0.83	2.91	0.65
**Men**						
20–25	–	–	4.34	0.41	4.13	0.50
40–45	4.57	0.68	3.86	0.49	3.33	0.68
60–65	4.05	0.65	3.79	0.39	2.93	0.90


The pattern in *f*_0_ observed from **Table 1** was supported by a 3 × 2 × 2 mixed analysis of variance with voice condition (young, natural, old) as the within-subject variable and speaker age group (40–45, 60–65 years) and sex (female, male) as the between-subjects variables. The analysis revealed main effects of voice condition and sex but no interaction effects. Hence, speakers did only to some extent manipulate *f*_0_ in directions corresponding to actual age related changes in *f*_0._ Speakers used higher *f_0_* (*M* = 166.19 Hz, *df* = 47.79) to sound younger compared with their undisguised voice (*M* = 156.40 Hz, *df* = 41.95) and lower *f_0_* to sound older (*M* = 145.20 Hz, *df* = 40.77, *F*[2,40] = 16.68, *p* < 0.001, *MSE* = 158.76, $\eta_{p}^{2}$ = 0.46, both differences were verified by a *post hoc* test using the Bonferroni correction, *p* < 0.05) which corresponds to the direction of actual *f_0_* change in female but not entirely in male speakers. As expected, the voices of female speakers (*M* = 193.75 Hz, *df* = 19.57) were higher-pitched than those of male speakers (*M* = 118.11 Hz, *df* = 15.88, *F*[1,20] = 105.67, *p* < 0.001, *MSE* = 974.66, $\eta_{p}^{2}$ = 0.84).

**FIGURE 1:**
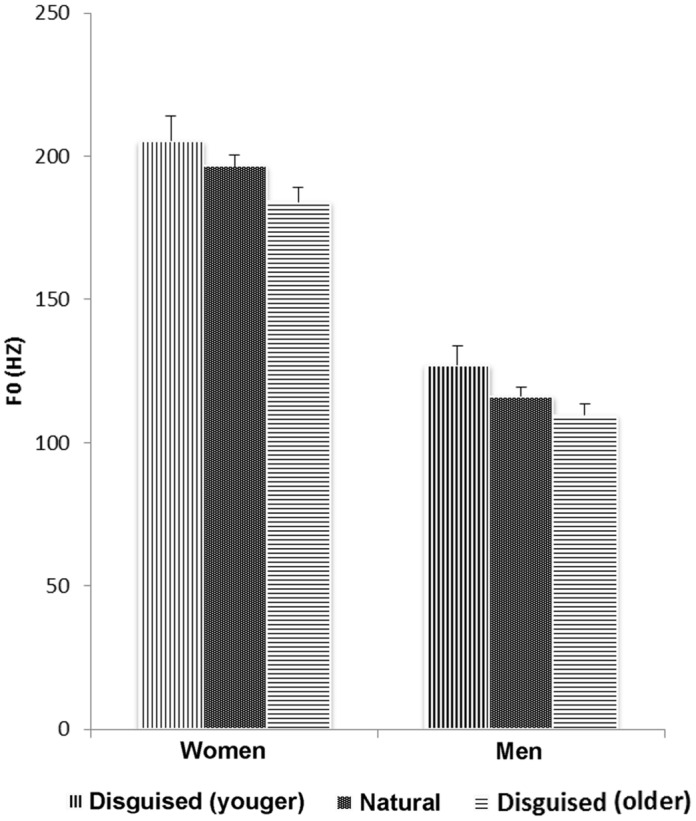
**Change in fundamental frequency (*f*_0_) when women and men disguise their voice.** Error bars indicate the standard error of the mean (SEM).

The results above were supported by a 2 × 3 × 2 ANOVA with voice condition (natural, older) as the within-subject variable and age group (20–25, 40–45, 60–65 years) and sex (female, male) as the between-subjects variables. The analysis again revealed a main effect of voice condition, (*F*[1,30] = 14.57, *p* = 0.001, *MSE* = 113.72, $\eta_{p}^{2}$ = 0.33) but no interactions. The speakers used a lower *f_0_* when disguised as old compared with the natural voices. Women’s *f_0_* were also higher than those of men (*F*[1,30] = 194.37, *p* < 0.001, *MSE* = 553.80, $\eta_{p}^{2}$ = 0.87). Neither analysis yielded a main effect of age group (**Figure 1**).

#### Speech Rate

Mean and standard deviation of speech rate for women and men over voice conditions and age groups are shown in **Table 1**. Both female and male speakers spoke faster when disguised as younger and slower when disguised as older. This was first confirmed by a 3 × 2 × 2 mixed analysis of variance with voice condition (young, natural, old) as the within-subject variable and speaker age group (40–45, 60–65 years) and sex (female, male) as the between-subject variables. The analysis demonstrated a main effect of voice condition. Speakers spoke faster (*M* = 4.37 syll/s, *df* = 0.61) when disguised as younger as compared with their natural voices (*M* = 3.82 syll/s, *df* = 0.50) and slower when disguised as older (*M* = 3.12 syll/s, *df* = 0.76, *F*[2,60] = 47.68, *p* < 0.001, *MSE* = 0.189, $\eta_{p}^{2}$ = 0.71, both differences were verified by a *post hoc* test using the Bonferroni correction, *p* < 0.05). There was also a main effect of age such that speakers aged 40–45 years spoke faster than speakers aged 60–65 years, (*F*[2,30] = 3.98, *p* < 0.029, *MSE* = 0.791, $\eta_{p}^{2}$ = 0.21). Finally, there was also an interaction between voice condition and age group (*F*[2,60] = 5.32, *p* = 0.001, *MSE* = 0.184, $\eta_{p}^{2}$ = 0.26) indicating that speakers aged 40–45 years increased there speech rate more when attempting to sound younger compared to the speakers 60–65 years old.

**FIGURE 2:**
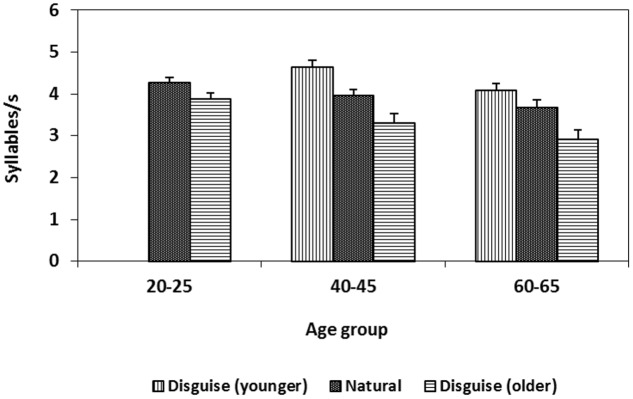
**Change in speech rate (syllables/s) when speakers from three age groups disguise their voice.** Error bars indicate the standard error of the mean (SEM).

The results were further supported by a 2 × 3 × 2 mixed ANOVA with voice condition (natural, older) as the within-subjects variable, and age group (20–25, 40–45, 60–65 years) and sex (female, male) as between-subjects variables. There was a main effect of voice condition, (*F*[1,30] = 45.07, *p* < 0.001, *MSE* = 0.142, $\eta_{p}^{2}$ = 0.60) but no significant interactions. Speakers spoke slower when attempting to disguise their voice to sound 20 years older (*M* = 3.38 syll/s, *df* = 0.77) compared with no disguise (*M* = 3.97 syll/s, *df* = 0.56). Thus, speakers manipulated speech rate in the direction corresponding to actual age related change. Inclusion of the younger age group led to a main effect of age group (*F*[2,30] = 6.18, *p* = 0.006, *MSE* = 0.613, $\eta_{p}^{2}$ = 0.98). Speakers aged 20–25 years spoke faster (*M* = 4.09 syll/s, *df* = 0.42) than speakers aged 60–65 years (*M* = 3.29 syll/s, *df* = 0.69) as confirmed by a *post hoc* test using the Bonferroni correction (**Figure 2**).

In sum, subjects increased *f*_0_ and speech rate as compared with their natural voice when trying to sound younger, whereas they decreased *f*_0_ and speech rate when trying to sound older. No interaction between *f*_0_ and age or between speech rate and age could be verified. The change in speech rate was larger than the change in *f*_0_, as indicated by the effect sizes. In addition, differences in speech rate were found between the speakers as a function of their age. No effects of chronological age were revealed for *f*_0,_ although *f*_0_ was sensitive to the sex of the speaker.

## Study 2

The purpose of the second study was to investigate how successful the voice disguise from Study 1 was by asking naïve listeners to estimate the speakers’ age. The study of Lass et al. (1982) was extended by including voices from three age groups. We expected to replicate Lass et al.’s (1982) finding that young speakers are able to manipulate their voices to sound older. However, we believed that middle aged and older speakers would be less successful than young speakers to disguise their voices to sound younger or older. Because *f*_0_ are in another range for women than for men, we also asked whether women and men were equally good at modifying their voices to sound a different age. Finally, it was asked if disguising the voice to sound younger was as effective as disguising the voice to sound older.

### Method

#### Participants

Sixty students (47 females and 13 males) with Swedish as their native language participated in the experiment. The students’ mean age was 26.1 years (*SD* = 5.76, range = 18–41 years). As in Study 1, voluntary, informed consent was provided by the participants and the ethical guidelines of the American Psychological Association were followed.

#### Material and Procedure

The voices described in Study 1 were presented to the listeners in a laboratory setting through headphones. The listeners were instructed to estimate the age (in years) of each speaker they heard in the headphones and write their estimates in a protocol. A pause of 10 s followed each voice presentation. Backtracking was not possible. The session lasted about 20 min. Participation was rewarded with a movie ticket.

The listeners were randomized into three listener groups. Each group listened to 12 neutral speech samples that were produced by two female and two male speakers from each age group, 12 speech samples disguised to sound older that were produced by two female and two male speakers from each age group, and eight speech samples disguised to sound younger that were produced by two female and two male speakers from each of the two older age groups (see **Table 2**). Hence, each participant listened to and estimated the age of 32 voices. The speech material differed between the listener groups with respect to in which voice condition a voice was presented. A listener heard a voice in only one voice condition. The speech samples were presented in a randomized order within each listener group with a 10 s pause after each voice.

**Table 2:** Illustration of the composition of speech samples (disguised to sound younger/natural/disguised to sound older) in three different listener groups.

Listener group	*1*	*2*	*3*
Speaker 1♀^∗^		natural	older
Speaker 2♀^∗^	older		natural
Speaker 3♀^∗^	natural	older	
(**…**)			
Speaker 7♂^∗^		natural	older
Speaker 8♂^∗^	older		natural
Speaker 9♂^∗^	natural	older	
(**…**)			
Speaker 13♀^∗∗^	younger	natural	older
Speaker 14♀^∗∗^	older	younger	natural
Speaker 15♀^∗∗^	natural	older	younger
(**…**)			
Speaker 19♂^∗∗^	younger	natural	older
Speaker 20♂^∗∗^	older	younger	natural
Speaker 21♂^∗∗^	natural	older	younger
(**…**)			
Speaker 34♂^∗∗∗^	younger	natural	older
Speaker 35♂^∗∗∗^	older	younger	natural
Speaker 36♂^∗∗∗^	natural	older	younger


*^∗^20–15 years, ^∗∗^40–45 years, ^∗∗∗^60–65 years.*

#### Analyses

The statistical analyses were conducted in SPSS 22.0 using repeated analysis of variance (ANOVA). *Post hoc* analyses were computed using the Bonferroni correction and the level of significance was set at 0.05. Because the design did not include young speakers attempting to sound younger, there were three voice conditions for only two age groups (40–45, 60–65 years). As in Study 1, two analyses were first performed. The first was a within-subject ANOVA that included three voice conditions (young, natural, old) and two speaker age groups (40–45, 60–65 years). The second ANOVA was a within-subject ANOVA consisting of two voice conditions (natural, old) and three speaker age groups (20–25, 40–45, 60–65 years). Speaker sex was included as a third (within-subjects) factor in both analyses. When Mauchly’s test indicated deviance from conventional sphericity assumptions, Greenhouse–Geisser adjusted degrees of freedom (df) were used, although df are reported in integers for readability. To ensure that the effect of voice disguise on perceived age in Study 2 was not caused by only a few speakers manipulating their voices, two additional repeated measures ANOVAs were computed across the target voices (younger, natural, and older) in F2 analyses. Accuracy rates were based on the unsigned deviation of estimated age from chronological age which is a more direct measure of exactness than correlations between chronological and estimated age. Linear regression analyses were computed to investigate how much of the variance in estimated age *f*_0_ and speech rate accounted for in the three voice conditions. Sex was also included in the model because it’s strong relatedness to *f*_0_.

### Results and Discussion

**Table 3** show mean and SD of estimated age of women and men over voice conditions and age groups. The descriptive data from the upper part of **Table 3** indicate that the ages of the speakers were estimated in the attempted direction. Speakers disguised to sound older were estimated as older and speakers disguised to sound younger were estimated as younger compared to the age estimates of their natural voice, although the changes in age estimates between conditions were small.

**Table 3:** Mean and standard deviations of estimated age and accuracy (years) of women and men over disguise conditions and age groups in Study 2.

	Disguise (younger)		Natural		Disguise (older)	
			
	*M*	*SD*	*M*	*SD*	*M*	*SD*
**Estimated age**		
**Women**						
20–25	–	–	27.32	6.25	28.93	8.09
40–45	35.33	6.61	38.70	6.40	40.43	7.22
60–65	52.06	7.96	53.89	7.56	57.93	10.11
**Men**						
20–25	–	–	31.46	6.75	33.89	7.95
40–45	41.23	9.61	43.52	8.22	45.08	9.89
60–65	50.97	8.05	52.90	7.33	59.50	9.49
**Bias (signed deviation: estimate minus target age)**		
**Women**						
20–25	–	–	4.65	3.60	6.27	3.70
40–45	-5.33	3.42	-1.80	3.07	-0.07	4.79
60–65	-11.27	4.12	-9.44	4.25	-5.40	5.70
**Men**						
20–25	–	–	5.35	3.13	9.56	4.10
40–45	-1.10	4.79	1.18	4.18	2.74	4.59
60–65	-11.20	4.10	-9.27	2.97	-2.67	4.34
**Accuracy (unsigned deviation: estimate minus target age)**		
**Women**						
20–25	–	–	6.17	1.60	8.00	3.08
40–45	9.30	2.32	8.55	1.95	7.92	2.45
60–65	13.56	2.83	11.54	3.23	11.02	3.23
**Men**						
20–25	–	–	4.80	4.04	9.86	4.04
40–45	9.27	3.00	7.40	3.10	9.04	2.55
60–65	12.37	3.39	10.80	2.16	8.82	3.36


**N* = 60.*

Two commonly used measures of exactness, *bias* and *accuracy* (e.g., Vestlund et al., 2009; Skoog Waller et al., 2015), are also included in **Table 3**. The bias measure is based on the signed deviation of the estimate minus the speaker’s chronological age. As positive and negative values cancel each other when added to a mean, this measure reflects general trends of overestimations and underestimations. **Table 3** shows that the age of speakers aged 60–65 years were underestimated in all conditions. Moreover, underestimations were larger and more frequent when the speakers were disguised as younger and overestimations occurred for the youngest age groups and for men 40–45 years when disguised as older.

The accuracy measure in **Table 3** adds the unsigned deviations from the speakers’ chronological age and thus gives a direct measure of how accurate age estimates are in general. Accuracy was expected to be highest in the natural condition and this was also the case for 20–25 years old speakers and for men aged 40–45 years. However, because of the frequent underestimations of the age of older speakers, accuracy was actually highest for men 60–65 years when attempting to be older and to the two oldest age groups and for women aged 40–45 years when disguised as older (**Table 3**).

A 3 × 2 × 2 within-subject ANOVA with voice conditions (young, natural, old), speaker age group (40–45, 60–65 years) and speaker sex (female, male) showed a main effect of voice condition in the expected direction. Voices were estimated as younger when the speakers were instructed to sound 20 years younger (*M* = 44.90 years, *df* = 8.05) compared with their natural voice (*M* = 47.25 years, *df* = 7.38) and voices were estimated as older when disguised to sound 20 years older (*M* = 50.74 years, *df* = 9.18, *F*[2,94] = 31.29, *MSE* = 83.30, *p* < 0.01, $\eta_{p}^{2}$ = 0.35). *Post hoc* test using the Bonferroni correction revealed significant differences between all three voice conditions (*p* < 0.05). There was also an expected effect of age group: older voices were estimated as older (*M* = 54.54 years, *df* = 8.42) compared with younger voices (*M* = 40.72 years, *df* = 7.99, *F*[1,59] = 482.28, *MSE* = 71.35, *p* < 0.01, $\eta_{p}^{2}$ = 0.89). A main effect of sex indicated that men (*M* = 48.87 years, *df* = 8.77) were estimated as being older than women (*M* = 46.39 years, *df* = 7.64, *F*[1,59] = 21.58, *MSE* = 51.04, *p* < 0.01, $\eta_{p}^{2}$ = 0.27). An interaction was noted between age group and sex, (*F*[1,59] = 36.85, *MSE* = 34.19, *p* < 0.01, $\eta_{p}^{2}$ = 0.38), indicating that it was only in the ages 40–45 years that men were estimated as older than women. No other interactions were significant. The F2 analysis over the 24 voices in three conditions (young, natural, and old) indicated that the result is generalizable across voices (*F*[2,46] = 19.43, *MSE* = 10.65, *p* < 0.01, $\eta_{p}^{2}$ = 0.46).

The results above were confirmed by a 2 (voice condition: natural, older) × 3 (age group: 20–25, 40–45, 60–65 years) × 2 (sex: female, male) within-subject ANOVA. The main effects of voice condition (*F*[1,59] = 26.84, *p* < 0.001, *MSE* = 60.25, $\eta_{p}^{2}$ = 0.31), age group (*F*[2,92] = 405.67, *p* < 0.001, *MSE* = 125.19, $\eta_{p}^{2}$ = 0.87, *post hoc* test using the Bonferroni correction indicated significant differences in estimated age between speakers of all three age groups, *p* < 0.05) and sex (*F*[1,59] = 54.57, *p* < 0.001, *MSE* = 33.54, $\eta_{p}^{2}$ = 0.48) remained, as did the interaction between age group and sex (*F*[2,118] = 9.50, *p* < 0.001, *MSE* = 39.92, $\eta_{p}^{2}$ = 0.14). Male voices were now estimated as older than female voices both for the 20–25-year-old speakers that were added in this analysis (male voices *M* = 32.7 years, *df* = 4.99 vs. female voices *M* = 28.1 years, *df* = 5.88) and for the 40–45-year-old speakers (male voices *M* = 43.3 years, *df* = 6.14 vs. female voices *M* = 38.2 years, *df* = 4.09). This pattern was confirmed by paired sample *t*-tests. No difference was found in estimated age between women and men 60–65 years old. That the female voice experiences more salient vocal changes in later adulthood than the male voice is supported by findings from earlier acoustic studies (Decoster and Debruyne, 1997).

**FIGURE 3:**
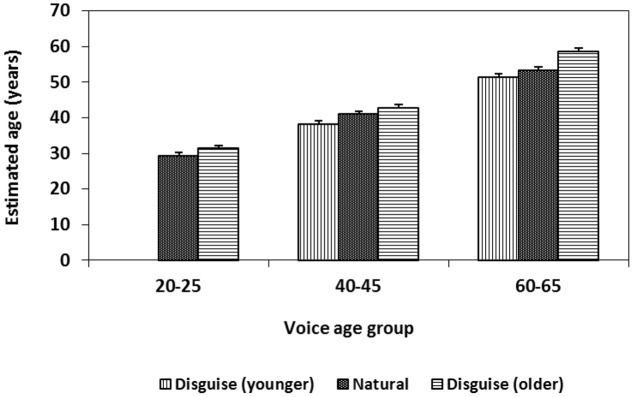
**Effects of voice disguise to sound younger or older than normal on perceived age over three age groups of speakers.** Error bars indicate the standard error of the mean (SEM).

In addition, the last ANOVA involving three age groups revealed an interaction between age groups and voice condition, *F*(2,118) = 4.86, *MSE* = 54.18, *p* = 0.01, $\eta_{p}^{2}$ = 0.076. Although age was estimated as higher when the speakers’ voices were disguised as older for all three age groups when compared with the natural condition, this difference was especially pronounced for estimates of voices in the oldest age group (**Figure 3**). The main effect of voice condition support our expectation that young speakers (as well as middle aged and older speakers) are able to manipulate their voice to sound older. However, the interaction between voice condition and age group goes in the opposite direction from the expectation that middle aged and older speakers would be less successful than young speakers to disguise their voices to sound younger or older.

To directly test whether voice disguise of the speaker to sound younger was as effective as voice disguise to sound older the two change scores from the natural condition (to sound younger and to sound older) were computed for the 40–45- and 60–65-year-old speakers. A paired sample *t*-test revealed no difference in estimated age in speakers’ attempts to sound younger or older, (*t*[59] = 1.25, ns.). The F2 analysis over the 36 voices and two conditions (natural and old) indicated that the result is generalizable across voices (*F*[1,35] = 18.06, *MSE* = 10.81, *p* < 0.01, $\eta_{p}^{2}$ = 0.34).

As in other studies of age estimation by voice (Shipp and Hollien, 1969; Hollien and Tolhurst, 1978; Huntley et al., 1987; Braun, 1996; Braun and Cerrato, 1999; Brückl and Sendlmeier, 2003; Harnsberger et al., 2008; Skoog Waller et al., 2015), the age of young speakers was overestimated and the age of old speakers was underestimated. These estimation biases result in a cluster around the population mean. Over- and under-estimations from the natural condition in the present study were confirmed by one-sample *t*-tests showing that the estimated mean age for speakers aged 20–25 years (*M* = 29.4 years, *df* = 5.95) was significantly higher than the speakers’ chronological age (*M* = 23.4, *df* = 1.19, *t*[59] = 7.82, *p* < 0.01), and the estimated mean age for speakers aged 60–65 years (*M* = 53.4 years, *df* = 6.45) was significantly lower than the speakers’ chronological age (*M* = 62.7, *df* = 1.87, *t*[59] = -11.14, *p* < 0.01). No difference was found between estimated age and chronological age for speakers aged 40–45 years. Given these estimation biases, it seems likely that disguise toward middle age would be greater than disguise in the other directions (younger, older). However, this hypothesis was not confirmed as the interaction between voice condition and age group pointed in the other direction. Neither did the results support the expectation that older speakers would be less able to disguise their age as speakers aged 60–65 years were particularly successful in manipulating their voices to sound older (**Figure 3**). Hence, the interaction between voice condition age group was not caused by a regression toward the mean.

### Predicting Age Estimates from *f*_0_ and Speech Rate

A hierarchical regression analysis was computed to investigate how *f*_0_ and speech rate (from Study 1) were related to estimated age (from Study 2). Considering the non-linear relation between *f*_0_ and age described by Linville (1996), the linearity of *f*_0_ and speech rate in relation to estimated age in the present data was first explored. Although a cubic transformation did equally well for speech rate, no transformation was found (logarithmic, inverse, quadric, cubic, S, logistic, growth, exponential) to beat a simple linear relation for either *f*_0_ or speech rate in terms of explained variance (*R*^2^) in estimated age. The correlation between *f*_0_ and speech rate was low (*r* = 0.075, *N* = 96, *p* = 0.466), hence multicollinearity was no problem.

Because of different levels and different developmental trajectories of *f*_0_ in women and men, speaker sex was included in a first block. In this block, voice condition was also included as attempts to sound younger or older might affect the influence of *f*_0_ and speech rate on estimated age. In the second block *f*_0_ and speech rate were entered together and estimated age served as dependent variable. This model accounted for 24.3% of the variance (adj *R^2^* = 0.201) in estimated age. The first block with speaker sex and voice conditions made no significant contribution to estimated age (*R*^2^ = 0.037, adj *R*^2^ = 0.006, *p* = 0.322). Speech rate was the only parameter in the second block that reached significance (*p* < 0.001). See **Table 4** for regression coefficients and *p*-values of block 2 (all variables in block 1 were dummy coded). In conclusion, the participants in Study 2 relied strongly on speech rate but used little information from *f*_0_ when estimating speaker age from voice. Information from speech rate was used regardless of speech condition.

**Table 4:** Predicting estimated age from *f*_0_ and speech rate.

Variable	*B*	*SE B*	β	*t*	*p*
*f*_0_	-0.083	0.054	-0.318	-1.540	0.127
Speech rate	-7.534	1.631	-0.497	-4.618	<0.001


*Sex and voice condition in block 1 were dummy coded and these coefficients are therefore not reported. They made no significant contribution. *N* = 96.*

## General Discussion

One purpose of this research was to investigate how people manipulate their voices when they attempt to sound younger or older than their chronological age. Another aim was to evaluate the effectiveness of such speech manipulations. The results indicate that speakers increase *f*_0_ and speech rate when trying to sound younger and decrease *f*_0_ and speech rate when trying to sound older. This strategy was applied regardless of speaker sex or age. The strategy was effective in that voices in the two disguised voice conditions obtained age estimates in the attempted direction. This finding held for both female and male voices, and there was no difference in effectiveness between voice disguise to sound younger and voice disguise to sound older. An interaction was found between vocal disguise and age group, such that speakers 60–65 years old were more successful in sounding older than speakers from the other age groups. However, this interaction is probably of little practical importance in that few 60-year-olds would gain much from appearing older, and in absence of other interactions, we conclude that the effect of voice disguise is robust but the effect on age estimations is rather small, typically varying from 2 to 4 years.

Although speakers made linear changes in both *f*_0_ and speech rate when trying to sound younger and older, it was speech rate that explained the variance in estimated age (around 20%). Hence, much variance remains to be explained and a limitation of the present paper was that only two (although generally acknowledged as the most important) speech parameters were investigated. However, the variation in age estimates of voice is typically high; the deviation from chronological age in estimations of speaker age is generally 7–11 years (Krauss et al., 2002; Schötz, 2006; Hughes and Rhodes, 2010). Given the non-linear relation of *f*_0_ to age and the different developmental trajectories for women and men described by Linville (1996), *f*_0_ is probably a hard to get cue to speaker age, and it would be inefficient to try to extract age relevant information from *f*_0._ Yet, one could still ask why the speakers changed *f*_0_ and not only speech rate when attempting to sound younger or older? A simple answer would be that it is just a side effect of intentionally changing the speech rate, resembling van Son and Pols (1990) finding that the vowel frequency raised when the speech rate increased. However, we also asked the speakers of the present study how they adjusted their voice, and about half of them mentioned spontaneously that they raised their voice when sounding younger. This opens for that the change in *f*_0_ was in some part intentional and probably also controlled. We therefore speculate that the speakers in this study modified their voices according to their stereotypes of how young and old voices sound. More research on stereotypes of vocal aging is needed and so are studies on which speech parameters we can and do change intentionally.

The small effect of vocal age disguise on perceived age corroborates previous findings (Lass et al., 1982). Considering the task in the current study was to change the voice to sound 20 years younger or older, a change of 2–4 years is modest. For instance, such a small change is probably of no relevance for criminals trying to disguise their true age. However, the instruction to change the voice 20 years in one direction or the other may not have been taken literally by the speakers. Most of us are unfamiliar to the idea of being able to change our vocal age with that type of precision. Instead, it is likely that the instruction was interpreted as to modify the voice to sound “much younger” and “much older.” Still, the facts remain that 2–4 years is not very much.

Another issue concerns the speech material. The present experiments were based on short passages (9–12 s) of read speech. Read speech allows for better linguistic control than spontaneous speech. In addition, it makes speech parameters (such as *f*_0_ and speech rate) directly comparable across speech samples. On the other hand, spontaneous speech contains other information, including word choice, choice of grammatical constructions, prosody and fluency, which probably displays important age cues as well. Previous research (Schötz, 2005; Skoog Waller et al., 2015) have also found that age estimates from a person’s voice are more accurate when based on spontaneous speech than on samples of read speech. Thus, spontaneous speech offers more age related parameters to vary compared to read speech. However, weather this is to the advantage of the speaker wanting to disguise her or his true age, or to a listener trying to estimate the age of the speaker is hard to tell. This notion has applications for ear witness confrontations where the witness is asked to identify a perpetrators voice, often from speech samples of read speech. Future research should study how age estimation and identification is affected by speech material.

One primary purpose of voice disguise for criminals is to aggravate identification. It might be that small effects of disguising age by voice, as those found in the present study, is effective for that intention, especially if the heard voice is an unfamiliar one. The vocal basis of age perception and the way in which different factors influence that process is also of interest in acting where age is often an essential dimension in the role played. Some parts will demand from the performer to act another age partly through vocal manipulation. Findings on how to successfully influence perceived age in either direction is therefore valuable in theater and film. However, it is important to keep in mind that the perceived effect in this study was rather small. That the speakers were not able to disguise age more effectively may depend on physical factors or more constant voice parameters which cannot easily be disguised by the speakers. It would be of great value for crime investigations to identify voice parameters that are resistant to disguise. The elimination of easy changeable parameters such as *f*_0_ and speech rate is also one step toward this end.

## Author Contributions

SS and ME designed the experiment. SS performed the data collection. SS and ME analyzed the data. SS and ME wrote the manuscript.

## Conflict of Interest Statement

The authors declare that the research was conducted in the absence of any commercial or financial relationships that could be construed as a potential conflict of interest.

## Footnotes

1 www.audacity.sourceforge.net

2 www.praat.org
